# Digital Technologies Supporting Person-Centered Integrated Care – A Perspective

**DOI:** 10.5334/ijic.3051

**Published:** 2017-09-25

**Authors:** John Øvretveit

**Affiliations:** 1 Karolinska Institutet Stockholm, SE

**Keywords:** Health information technology, integrated care, clinical care coordination, communication, quality

## Abstract

Shared electronic health and social care records in some service systems are already showing some of the benefits of digital technology and digital data for integrating health and social care. These records are one example of the beginning “digitalisation” of services that gives a glimpse of the potential of digital technology and systems for building coordinated and individualized integrated care. Yet the promise has been greater than the benefits, and progress has been slow compared to other industries. This paper describes for non-technical readers how information technology was used to support integrated care schemes in six EU services, and suggests practical ways forward to use the new opportunities to build person-centered integrated care.

## Introduction

This paper uses the non-technical term “digitalisation” to describe the introduction of digital devices and systems for collecting and using data for many purposes. It concentrates how we can best use such data and systems to enable coordination of clinical activities between beneficiaries and providers and between providers. It also considers how these data, generated in the course of clinical care and of beneficiaries lives, can be used to inform planning and management decisions about integrated care and used for research. The author’s perspectives and values are those of a technology enthusiast, a former clinician and now researcher, but also with a concern about the possible harm to personal care and relationships that could come from unskillful design and inappropriate use of the data and of these powerful technologies.

## The potential and the progress

Most clinicians, leaders and researchers have some awareness of the potential of IT for enhancing health and social care and its coordination, but also of the dangers and time distractions of under-designed systems. Many note the slow progress in using IT effectively to enable coordination for of care for service beneficiaries (patients/clients). Care providers need information about a beneficiary’s wants and needs, and about what other providers are doing if they are to adjust their help to compliment the help of others: the “help of others” includes a beneficiary’s self-help and help of their close-carers. Beneficiaries often assume clinicians and care workers have access to all the necessary information about and for their care. Often it is beneficiary’s or close-carers who are the integrators of care, and can gain from knowing who else is involved, what they are doing, and from using IT tools to articulate their need and to follow their progress. In short, efficient integrated care is not possible without communication and information, and IT holds great promise for improving the quality of integrated care, for reducing costs and also making possible innovative models of IC.

As regards structures for integrated care, rather than by forming one fully integrated delivery system, more services are combining to build networks of independent organisations [1]. In other industries the “one organisation model” of integrated production is being replaced or “disrupted” with integrated supply chain approaches because the lower cost and sophistication of IT now makes this possible and because of the focus but coordination allowed by this network form [2,3]. This experience and the research from other industries shows ways to build new models of integrated care that IT and the internet makes possible which allow coordination but also a degree of autonomy for the networked units.

Another use of IT by industry that is relevant to integrated care is to create new and more efficient ways to interact with customers. This includes facilitating customer-customer interaction (peer to peer support), as well as gathering and using customer data for better customer management, prediction and planning [4]. In health and social care, modern person-centered integrated care (“PCIC”) recognises that most care is self-care and by close-carer family members and friends, and that the person must be at the center of the integration model, with providers supporting them and their close-carers [5,6]. IT enables services to transition to this model, as well as to bring new and more innovative ways of providing care: the principle of “move data not the patient” is useful for considering ways for reducing unnecessary and unwanted visits.

## IT to support integrated care in Europe: six cases

IT is increasingly used as one component of integrated care models for different patient groups: for example adding a smart phone app for beneficiaries to communicate with their care manager or a remote monitoring device. A second way is to use IT as the basis for a completely new way of providing integrated care – this is less common. To date, most published empirical reports are of IT systems that enable integration but involve considerable investment and long term development, and are mostly from the USA and possibly not relevant to many parts of Europe.

As part of the European Integrate project [7] a comparative case study was performed in 2015–2016 of the use of IT to support six different integrated care schemes: two in Sweden, two in the Netherlands, one in Spain and one in Germany [8]. The six schemes were for different beneficiary groups: people with mental health challenges, diabetes, and frail older people. The case studies gave an empirical “snapshot” of the IT systems supporting the integration, as well as of staff perceptions of the strengths, weaknesses and needed improvements.

The IT systems described in these cases studies reflected the professional and organisational fragmentation of the larger care systems of which they were part. The schemes sought to build integrated care for specific beneficiary groups, one connection at a time, and without radical restructuring and change. Information systems had been designed to serve individual professionals and separate services and were not very effective for supporting person-centered integrated care. The integrated care schemes had to work with the inherited buildings- and profession- based information systems and try to use digital technologies to improve communications.

Overall, the study found a limited use of IT to help integration in the six cases. The main technology used was electronic medical records that gave providers a way to collect, store, and retrieve their own beneficiary records, as well as some limited electronic access to other information about the beneficiary. In four of the six cases, providers could not securely exchange beneficiary’s information with providers working for other organisations, and most relied on fax, telephone and face to face meetings to communicate and coordinate care. The improvement that all providers wanted was for their organisation’s system to be able to connect with other organisation’s systems so as to be able to communicate and share information. Beneficiaries in five of the six cases studied did not have access to, and could not contribute to the health or social care information held about them [8].

## Ways forward for developing IT for integrated care

Recognising that the above study, there are still tentative lessons that can be drawn about how to make more and better use of IT to build integrated care. These are discussed as ten recommendations in the full study report and summarised below to help others to consider their role in shaping the development of IT for integrated care [8].

### Enabling data exchange

To support integrated care, a necessary requirement of any IT system is to be able to receive and send data, and in a way which the data cannot be accessed by unauthorized parties. In the USA, changes to ensure “interoperability” between systems are being driven by government requirements for electronic medical records (EMRs) and by health information exchanges, but the influences in Europe are not as strong to ensure interoperability [9,10]. New technologies and software are reducing the costs and security risks which arise from ensuring system inter-operability for integrated care [11]. With more providers recording personal data about the beneficiaries in the same system, more attention needs to be given to designs which allows fast access to, or presentation of, information at the time and place it is needed, for example by records structured in a way that enable integrated care.

### Engaging beneficiaries and close-carers

For future integrated care to be more person-centered, IT systems will need to enable beneficiaries and close-carers to specify their needs and goals and to allow them and providers easily to monitor and review their care plans. A structured approach to care needs assessment and planning can be led by one care coordinator, but there are different models for how other care providers are to be involved. IT does not replace the need for agreement about the social and organisational arrangements for their involvement: making data accessible does not mean it is accessed or acted on. Beneficiary access to their records can be facilitated by IT, but ways to ensure the privacy of data about third parties recorded in the records will be needed as will careful design and testing to ensure that beneficiaries who are not technically competent can use of benefit [12].

### Co-Design

In the European case study above, many providers reported that their IT systems were general purpose systems and not designed to support their integrated care work. The systems tended to reinforce old models of care rather that “nudge” providers towards more beneficiary-centered and multidisciplinary working models. To be effective, future systems will need to involve users in their design and implementation and will need to balance standardization with local tailoring to advance the model of integrated care that is envisioned. This means selecting users carefully for the co-design work, and choosing skilled designers who understand clinical and social care practice and can lead a co-design process.

### Data for planning and management

The key to cost control is a tiered coordinating care system, giving different levels of care intensity for different beneficiaries, and selecting beneficiaries for each level, based on accurate data [13,14,15]. Many current information systems need to be developed to provide the managers who are planning coordinated care with information on which they can base proposals or plans for such services. In addition, information for operational management of coordinated care schemes can be presented in actionable ways, such as by “dash board” visual presentations [16]. These systems need to be able to predict beneficiaries at high risk of deterioration and to obtain timely data about those beneficiaries making frequent avoidable use of emergency rooms or experiencing avoidable admissions [17,13,14].

### Research into IT to support IC

Our case study came to a conclusion similar to that of another study that, *“the pace of eHealth research has generally not kept up with technological advances, and many of the current designs, methods and funding mechanisms are incapable of providing the types of rapid and relevant information needed for successful ehealth implementation”* [18]. More research is needed independently to assess the claims and promises of the IT industry: is new hardware and software reducing costs and enabling local tailoring of systems to specific integrated care schemes, as well as improving security with biometric authentication?

Perhaps the greatest limiting factors to developing systems to support integrated care are the lack of skills and investment to co-design the systems to advance the visions of person-centered integrated care which are emerging [6]. Researchers have a role to play in these developments and can also help to make the systems usable for research which is lower-cost and more timely [19].

## Conclusion

“It is too technical for me – leave it to the experts” is a common and understandable response. Yet researchers and practitioners are able to, and have a responsibility to shape the systems to support the individualised person-centered and coordinated care we are trying to build. Not by asking permission to be involved, but by making it a requirement that systems are co-designed with users who are enabled to take part in the development. More humane, person-centered and less wasteful care is possible with these technologies. Equally possible are costly and cumbersome systems that take time from patient care and are difficult to access, that misuse data, and that turn necessary person-to-person physical visits into a luxury for the wealthy. The future of integrated care is too important to be left to technicians and the IT industry.
